# PET-based molecular imaging in neurodegeneration and dementia: current and emerging radiotracers

**DOI:** 10.1093/radadv/umag046

**Published:** 2026-10-13

**Authors:** Nelson Gil, Priyanka Naik, Mary Ellen Koran, Daniel Silverman, Bashar Kako, John Chen

**Affiliations:** Department of Radiology, Massachusetts General Hospital, Harvard Medical School, Boston, MA 02114, United States; Department of Radiology, Massachusetts General Hospital, Harvard Medical School, Boston, MA 02114, United States; Department of Radiology, Mayo Clinic Arizona, Phoenix, AZ 85054, United States; Department of Molecular & Medical Pharmacology, David Geffen School of Medicine at UCLA, University of California, Los Angeles, CA 90095, United States; Department of Radiology, Massachusetts General Hospital, Harvard Medical School, Boston, MA 02114, United States; Department of Radiology, Massachusetts General Hospital, Harvard Medical School, Boston, MA 02114, United States

**Keywords:** neurodegeneration, Alzheimer’s disease, dementia, PET, amyloid, tau, neuroinflammation, synaptic density, parkinsonism, theranostics

## Abstract

Positron emission tomography (PET)-based molecular imaging is a clinically important tool for the evaluation of dementia and other neurodegenerative disorders, using radiotracers that target specific molecular and pathophysiological processes. Fluorodeoxyglucose brain PET has historically supported neurodegenerative disorder evaluation through detection of characteristic patterns of regional glucose hypometabolism. In recent years, the molecular imaging of protein aggregates has taken on an increasingly important role. This is evident with the widespread clinical adoption of amyloid PET and early use of tau PET to provide diagnostic and prognostic information for patients with suspected Alzheimer’s disease. Additional classes of radiotracers relevant to neurodegenerative diseases are under active investigation, including those targeting neuroinflammation, synaptic density, and α-synuclein aggregates. We review the major classes of PET radiotracers for neurodegenerative diseases, describing their molecular mechanisms, diagnostic performance, and their current and emerging clinical roles in dementia evaluation. Case examples for clinically used tracers illustrate imaging features relevant to diagnosis and characterization of prognosis and treatment effect. Finally, we discuss early developments and current challenges in the development of theranostic approaches and novel radiotracers for neurodegenerative diseases.

**Summary** 

This review summarizes the clinical applications of PET radiotracers for neurodegenerative diseases, including those targeting β-amyloid, tau, and the striatal dopaminergic system and explores the potential of emerging research radiotracers.

**Essentials** 

- Protein-specific radiotracers (β-amyloid, tau) complement metabolic FDG-PET, improving the clinical evaluation of dementia patients.
- While FDG-PET, β-amyloid, and tau are clinically used, research tracers targeting tau, neuroinflammation, and synaptic density offer promising tools for early disease characterization and prognosis.
- Sequential tracer use may enhance diagnostic and prognostic characterization of Alzheimer’s disease.
- Diagnostic performance, anatomic target distribution, and off-target binding of the tracers are summarized.

## Introduction

Neurodegeneration is defined as the progressive loss of neurons. Alzheimer’s disease (AD) is the most common neurodegenerative cause of dementia, accounting for 60%-80% of cases and typically causing progressive decline in cognition and memory.^1^ Parkinson’s disease (PD) is the second most common neurodegenerative disease and is clinically characterized by physical symptoms of bradykinesia and tremor or rigidity. Some patients may also experience cognitive symptoms in the setting of well-established PD, which is classified as Parkinson’s disease dementia (PDD). PD and PDD are possibly linked to Lewy bodies (α-synuclein) in the brain, the presence of which signifies Lewy body disease (LBD). Dementia with Lewy bodies (DLB) is also associated with Lewy bodies, but patients develop earlier cognitive impairment relative to Parkinsonism.^2^ These neurodegenerative diseases are an important cause of morbidity and mortality due to the increased life expectancy and aging populations in many countries.^3^

Precisely diagnosing dementia based on clinical manifestations alone is challenging, particularly in early stages like mild cognitive impairment (MCI), due to overlap with normal age-related memory loss.^4^ Furthermore, conventional anatomic brain imaging, for example, with volumetric brain MRI, generally detects dementia only after significant neurodegeneration and parenchymal atrophy has occurred. Currently available therapies for neurodegenerative diseases primarily target symptoms. Even in PD, in which there are multiple longstanding therapeutic options, patients generally progress despite treatment.^5^ Furthermore, although anti-amyloid immunotherapy represents a promising recent advance in AD treatment, these therapies currently slow its progression but are administered after symptom onset.^6^^,^^7^ Therefore, to enable early intervention when drugs are thought to be most effective, diagnostic tools must precisely detect neurodegenerative diseases as early as possible in their course.

PET-based molecular neuroimaging complements conventional brain MRI in the evaluation of patients with suspected neurodegenerative disease. By directly probing pathological processes at the molecular level, PET radiotracers can anatomically localize disease-associated neuropathologic changes before macroscopic, MRI-evident tissue loss occurs.^8^ In addition, PET radiotracers can be used as biomarkers to monitor treatment response. The most salient examples come from the current use of anti-amyloid therapies in AD such as lecanemab, where amyloid PET can track the patient’s disease burden and response to therapy. While most developments in PET-based neuroimaging have focused on AD, similar principles can apply to other neurodegenerative diseases, including PD and DLB.

In this review, we summarize PET radiotracers used to evaluate neurodegenerative diseases, including both those in routine clinical use and promising emerging agents based on research findings. We discuss tracers used to evaluate AD and PD, examine how PET imaging findings inform clinical dementia management and highlight the potential for developing theranostic approaches in the field.

## Mechanisms of PET radiotracers for neurodegeneration

Current and emerging radiotracers for PET imaging of neurodegenerative diseases generally target pathological processes that are precursors or consequences of neurodegeneration. The most widely studied examples are in AD (Figure 1, Table 1), where the leading hypothesis is expressed by the amyloid, tau, neurodegeneration (ATN) framework.^9^ This posits that the progressive deposition in brain tissue of extracellular β-amyloid (A) and later intracellular tau (T) proteins into neurotoxic aggregates results in neurodegeneration (N). Although the detailed mechanisms for how these protein aggregates induce neurodegeneration are unknown, they are in part thought to trigger neuroinflammatory responses that result in local tissue damage, neuronal death, and synaptic loss.^10^ In PD, PDD, and DLB, similar pathophysiology is proposed, involving the abnormal deposition of α-synuclein protein aggregates and consequent loss of neurons.^11^ These pathophysiologic cascades can be targeted by different PET radiotracers.

**Figure 1:**
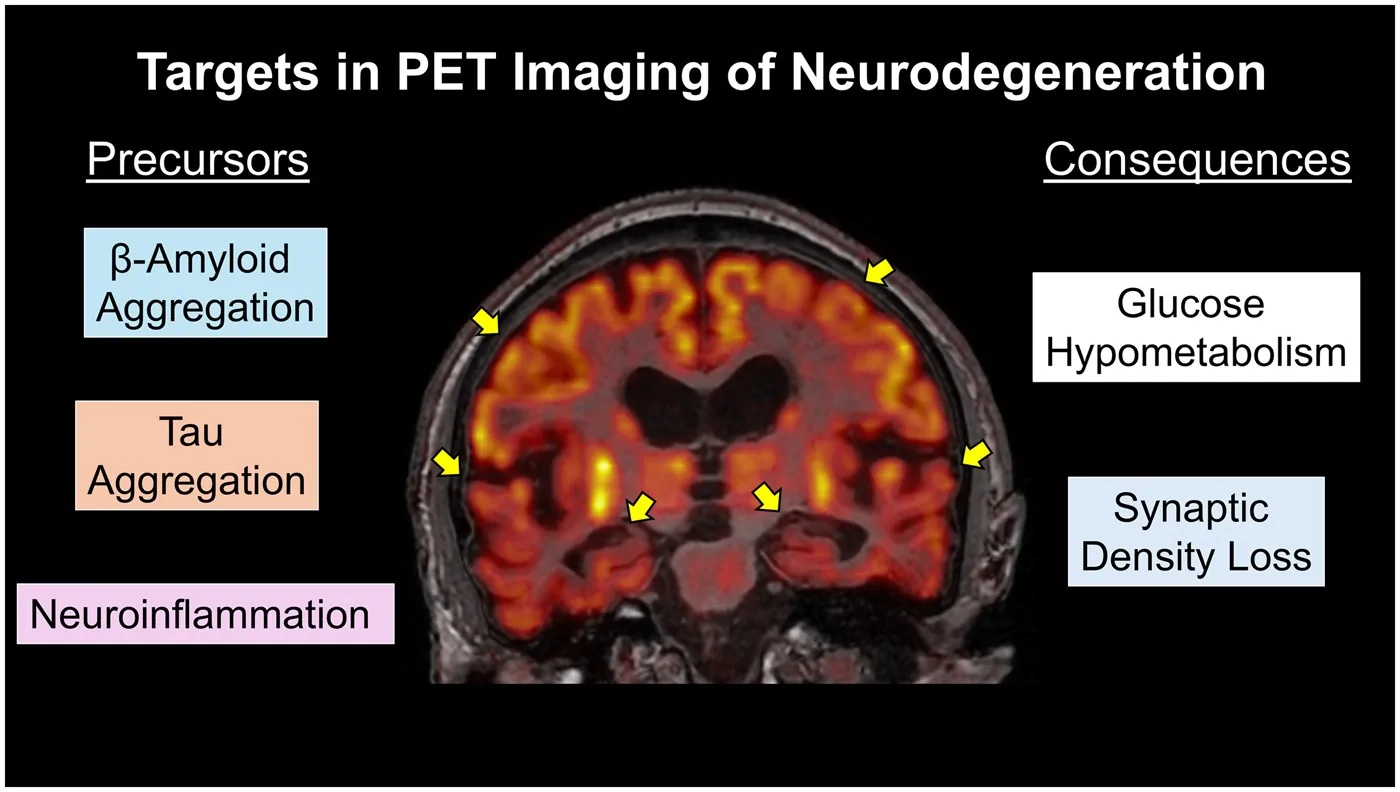
Representative pathological processes in Alzheimer’s disease (AD) that are targeted by current and emerging PET radiotracers. Neurodegeneration in AD can be evaluated using complementary structural and molecular imaging approaches, including T1-weighted volumetric MRI and PET. A coronal ^18^F-fluorodeoxyglucose (FDG) PET-MRI is shown, demonstrating cortical volume loss on MRI and regional glucose hypometabolism on FDG-PET, particularly in the temporoparietal regions and hippocampi (arrows). PET enables *in vivo* assessment of pathological processes that may precede, accompany, or result from neurodegeneration, including β-amyloid and tau aggregation, neuroinflammation, glucose hypometabolism, and synaptic density loss.

**Table 1:** Biological mechanisms assessed using PET radiotracers.

Physiologic/molecular target	Primary disease target	Typical radiotracer	Status in USA
**Glucose metabolism**	Various neurodegenerative diseases	^18^F-FDG	Clinical use
**Protein aggregation—β-amyloid**	Alzheimer’s disease	^18^F-florbetaben	Clinical use
**Protein aggregation—tau**	Alzheimer’s disease	^18^F-flortaucipir	Clinical use
**Dopaminergic neurotransmission**	Parkinson’s disease and Parkinson-plus syndromes	^18^F-DOPA	Research, emerging clinical, FDA approved
**Protein aggregation—α-synuclein**	Parkinson’s disease and Parkinson-plus syndromes	Experimental α-synuclein tracers	Research, not FDA approved
**Neuroinflammation**	Alzheimer’s disease	Experimental TSPO tracers	Research, not FDA approved
**Synaptic density**	Alzheimer’s disease	Experimental SV2A tracers	Research, not FDA approved

Abbreviations: FDG = fluorodeoxyglucose, SV2A = synaptic vesicle glycoprotein 2A, TSPO = translocator protein.

In general, clinically useful brain PET radiotracers are small molecules that incorporate fluorine-18 (^18^F). ^18^F’s 110-min physical half-life allows radiotracer distribution to sites without an in-house radiopharmacy.^12^ While carbon-11 (^11^C) is frequently used in research radiotracers, its clinical application is limited by a short (∼20 min) half-life that may preclude reaching steady-state equilibrium before significant signal decay. Consequently, a trend in the development of brain PET radiotracers has been to follow-up the discovery of ^11^C-based tracers with the development of ^18^F-based tracers. An example is β-amyloid PET imaging: the first ^11^C tracer, Pittsburgh compound B (^11^C-PiB), was reported in 2004, followed by the clinical approval of three ^18^F tracers.^12^^,^^13^ However, for some other molecular targets, the discovery of appropriate ^18^F-based tracers remains a barrier to clinical implementation.

## Fluorodeoxyglucose

Intracellular transport of ^18^F-fluorodeoxyglucose (FDG) molecules is proportional to glucose metabolism, leading to normal intense cortical and basal ganglia uptake on PET.^8^ Anatomical regions affected by neurodegeneration demonstrate relatively decreased FDG uptake. Different neurodegenerative diseases can result in characteristic patterns of regional decreased FDG uptake that can help increase the precision of a dementia diagnosis. Quantitative tools can compare the metabolic activity of different neuroanatomical regions in a patient to databases of age-matched cognitively normal controls and assign statistical *Z*-scores to aid visual assessment.^14^

The patterns of regionally decreased FDG uptake corresponding to different neurodegenerative diseases have been extensively reviewed previously.^8^^,^^14^ FDG brain PET’s most common use in dementia evaluation is to help differentiate AD from other dementias such as frontotemporal lobar degeneration (FTLD) or DLB, as well as to help identify atypical AD variants such as logopenic variant primary progressive aphasia (lvPPA) (Figure 2). FDG brain PET can also evaluate Parkinson-plus syndromes (motor features of Parkinsonism with additional atypical symptoms) including corticobasal degeneration (CBD) and progressive supranuclear palsy (PSP), as well as a recently-recognized dementia distinct from AD, limbic-predominant age-related TDP-43 encephalopathy (LATE).^14^ AD classically demonstrates regional hypometabolism in the parietal lobe, the posterior temporal lobe, the posterior cingulate gyrus, and the precuneus,^14^ though sufficiently advanced cases or AD variants may involve additional areas. On the other hand, FTLD primarily demonstrates hypometabolism in the frontal and anterior temporal lobes, which may be asymmetric. DLB demonstrates characteristic hypometabolism of the temporal, parietal, and occipital lobes with sparing of the posterior cingulate gyrus, resulting in the “cingulate island” sign. Finally, Parkinson-plus syndromes can have similar hypometabolism patterns to each other involving the frontoparietal cortex, basal ganglia, and thalamus, though CBD has been reported to often demonstrate asymmetric left-predominant findings.^15^ In addition, PD has been reported to be associated with hypometabolic patterns involving premotor and parietooccipital cortices,^15^ though due to the limited proven utility of FDG findings, nuclear imaging assessment of PD is more commonly done using other studies, including ^123^I-ioflupane (DaTscan) or, in a limited number of medical centers, ^18^F-DOPA-PET.^16^

**Figure 2:**
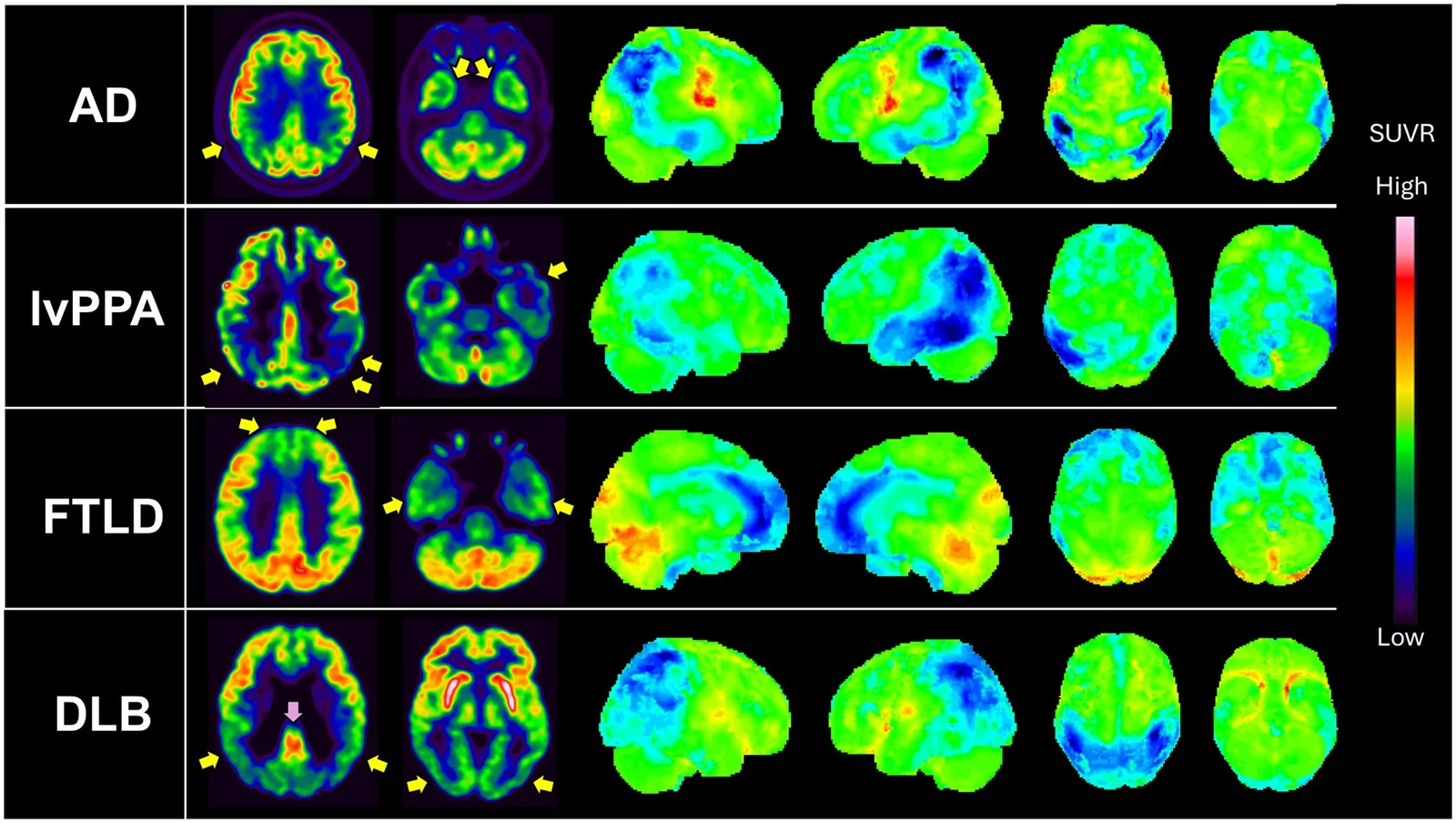
Patterns of cerebral hypometabolism as detected by FDG-PET standardized uptake value relative to the cerebellum (SUVR) in selected neurodegenerative diseases, displayed using Syngo.via (Siemens, Erlangen, Germany). In the 2 leftmost images of each row, areas of relative cortical hypometabolism in each condition are marked by yellow arrows. The 4 rightmost images in each row are stereotactic surface projections (SSPs) of FDG-PET data, with blue areas indicating relative hypometabolism and red areas indicating relative hypermetabolism. Alzheimer’s disease (AD) demonstrates relative cortical hypometabolism predominantly in the parietal and temporal lobes sparing the sensorimotor cortex, and logopenic variant primary progressive aphasia (lvPPA) demonstrates asymmetric, usually predominantly left-sided, temporoparietal hypometabolism. Frontotemporal lobar degeneration (FTLD) typically demonstrates hypometabolism in the frontal and anterior temporal lobes, and dementia with Lewy bodies (DLB) demonstrates hypometabolism in the occipital, parietal and temporal lobes, with notable sparing of the posterior cingulate (“cingulate island sign,” purple arrow). A qualitative scale bar is shown on the right.

FDG-PET has a long history in molecular neuroimaging and remains an important tool in the modern evaluation of dementia. For example, according to the American College of Radiology Appropriateness Criteria, the combination of qualitative and quantitative FDG-PET assessment resulted in a sensitivity of 95% for identifying AD versus other dementias, but a relatively lower specificity of 73%.^17^

## β-Amyloid

β-Amyloid is an extracellular variable-length peptide that, potentiated by certain biochemical modifications, can oligomerize and form aggregates that are the main components of the plaques detected on neuropathologic exams in AD.^12^ According to the ATN framework, β-amyloid plaque deposition in the extracellular space is the initial detectable pathophysiologic event that can lead to AD.^18^^,^^19^ Consequent neurodegeneration tends to affect specific neuroanatomical regions.

The detection of β-amyloid through brain PET serves as an *in vivo* assessment of neuropathological changes associated with AD. Currently, there are 3 ^18^F-based PET radiotracers approved for clinical use by the US Food and Drug Administration (FDA): ^18^F-florbetapir, ^18^F-flutemetamol, and ^18^F-florbetaben. All tracers demonstrate strong ability to detect brain amyloid plaques and minimal off-target binding to other proteins associated with neurodegeneration, as validated by PET-autopsy correlation studies: ^18^F-florbetapir resulted in sensitivity of 92% and specificity of 100%,^20^  ^18^F-flutemetamol a sensitivity of 81%-93% and specificity of 88%,^21^ and ^18^F-florbetaben a sensitivity of 98% and specificity of 89% (Table 2).^22^ Slight pharmacological differences between these 3 radiotracers result in minor relative advantages and disadvantages for each, as well as considerations when interpreting studies. For example, although ^18^F-florbetapir has among the strongest, most extensively validated PET-to-autopsy correlation data and relatively rapid imaging kinetics that may permit higher scan throughput, its relatively lower PET signal dynamic range combined with nonspecific white-matter retention can make subtle or borderline cases more challenging to interpret.^23^  ^18^F-flutemetamol is distinctive in that the striatum is explicitly incorporated into its qualitative interpretation criteria and may provide relatively strong striatal contrast, but it exhibits higher nonspecific white-matter uptake than ^18^F-florbetapir.^23^^,^^24^  ^18^F-florbetaben has a relatively flexible post-injection acquisition window and demonstrates high agreement among expert visual readers, but demonstrates similar to slightly higher white-matter binding compared to ^18^F-flutemetamol.^24^

**Table 2:** Specific properties of clinically used PET radiotracers to evaluate neurodegeneration.

Radiotracer	Target	Sensitivity/specificity for AD/PD	Potential advantage(s)	Potential disadvantage(s)
**^18^F-FDG**	Glucose metabolism	Sensitivity: 95%^17^ Specificity: 73%^17^	Easily accessible, general tracer with potentially relevant findings across different neurodegenerative diseases	Findings can be nonspecific for several neurodegenerative diseases
**^18^F-florbetaben**	β-Amyloid aggregates	Sensitivity: 92%^22^ Specificity: 100%^22^	Widest imaging window (35-130 min post-injection), high inter-rater reliability among the 3 FDA-approved brain amyloid PET tracers	Potentially higher white-matter binding compared to the other 2 FDA-approved brain amyloid PET tracers
**^18^F-florbetapir**	β-Amyloid aggregates	Sensitivity: 98%^20^ Specificity: 89%^20^	Slightly faster uptake kinetics compared to the other 2 FDA-approved radiotracers	Narrow imaging window (30-50 min post-injection), relatively lower dynamic range compared to the other 2 FDA-approved brain amyloid PET tracers
**^18^F-flutemetamol**	β-Amyloid aggregates	Sensitivity: 93%^21^ Specificity: 88%^21^	Can assess striatal β-amyloid uptake	High white-matter binding and lower gray-white-matter contrast compared to ^18^F-florbetapir
**^18^F-flortaucipir**	Tau aggregates	Sensitivity: 89.9%^35^ Specificity: 90.6%^35^	First clinically-approved tau PET tracer	Limited assessment of the medial temporal lobe because of off-target binding
**^18^F-MK-6240 (^18^F-florquinitau)**	Tau aggregates	Sensitivity: 83.8%^38^ Specificity: 90.2%^38^	Improved assessment of medial temporal lobe compared to ^18^F-flortaucipir	Off-target binding to multiple reported locations, including to the substantia nigra, globus pallidus, pineal gland, and possibly the meninges
**^18^F-DOPA (PD)**	Striatal dopaminergic system	Sensitivity: 95.4%^59^ Specificity: 100%^59^	High-resolution assessment of basal ganglia uptake	Higher cost compared to I-123 SPECT (DaTscan) limits adoption

Abbreviations: FDG = fluorodeoxyglucose, N/A = not available, PD = Parkinson’s disease.

Qualitatively, amyloid scans are read as visually positive or negative (Figure 3), with an adjunct quantitative uptake measurement such as the Centiloid score.^25^ A positive scan demonstrates increased cortical gray matter tracer uptake and loss of normal gray-white-matter contrast, whereas a negative scan preserves this contrast with nonspecific white-matter binding.^26^ Several qualitative signs have been described to help identify positive and negative scans, including the “summer/winter tree,” the “diamond sign,” and the “ridge sign” (Figure 4).^27^ Specific anatomical regions that differ slightly between tracers are evaluated for β-amyloid deposition: for ^18^F-florbetaben, interpretation evaluates the frontal and parietal cortices, the precuneus/posterior cingulate gyrus, and the lateral temporal lobe.^26^ Positive ^18^F-florbetaben scans can be further classified as moderately or diffusely positive using the brain amyloid plaque load (BAPL) scoring system^28^ (Figure 3). A specific challenge in qualitative interpretation arises when a patient has significant parenchymal volume loss, which can cause a PET to falsely appear negative (Figure 5A); in these cases, it may be helpful to co-register PET and available MRI data (or utilize a PET-MRI scanner) to help assess whether uptake on PET truly involves the cortex. Other challenges in interpretation include unexpected off-target uptake,^29^ which may even occur outside the brain parenchyma in the soft tissues of the head and neck (Figure 5B), and clinical judgment must be utilized to determine how to best perform a follow-up evaluation of such uptake.

**Figure 3:**
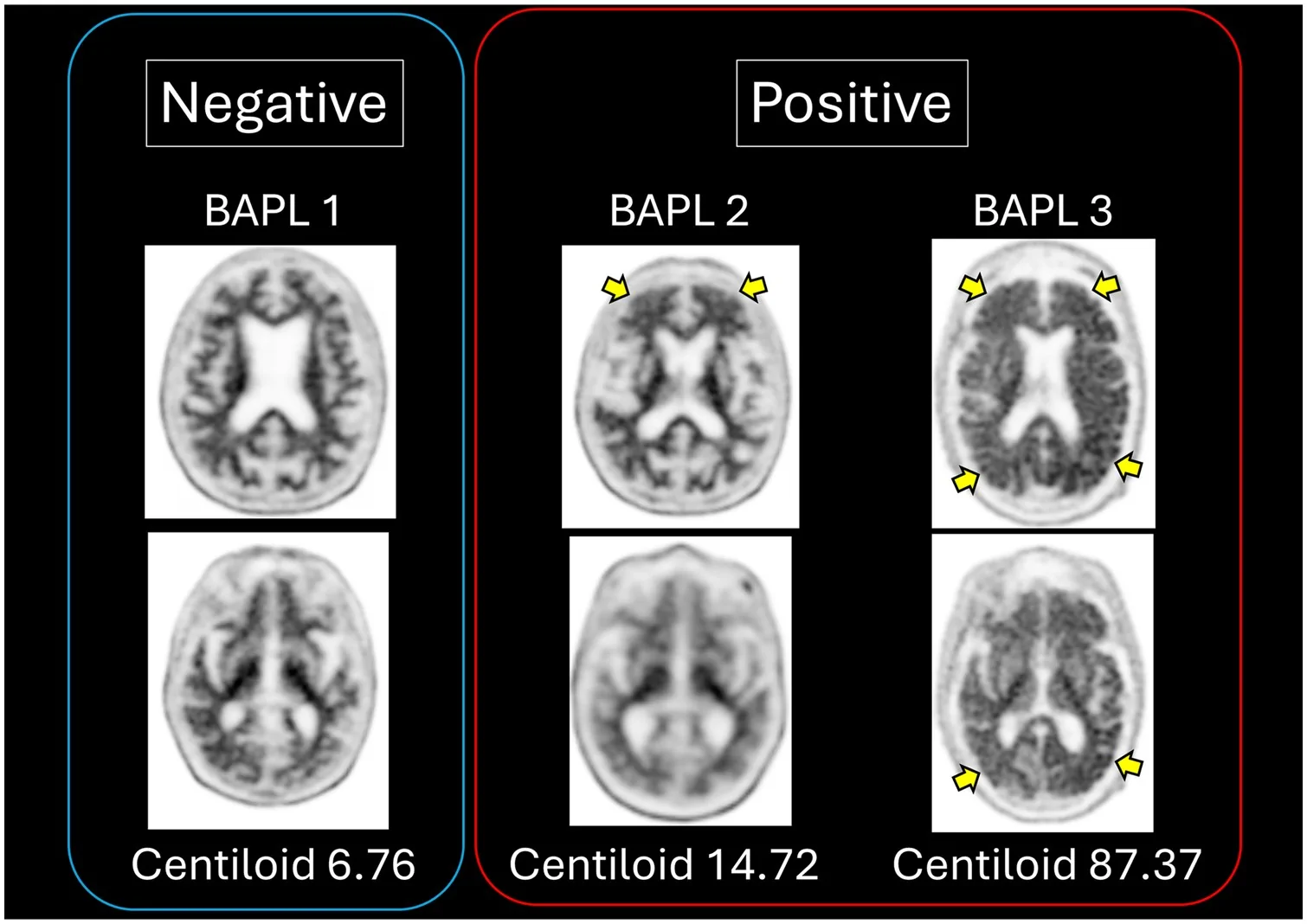
Negative and positive β-amyloid brain PET utilizing ^18^F-florbetaben, with subclassification based on the β-amyloid plaque load (BAPL) scoring system. Representative axial slices at the level of the lateral ventricles and ventricular atria are shown. Negative β-amyloid PETs only demonstrate radiotracer uptake in the white matter, with lack of uptake in the gray matter, corresponding to a BAPL 1 score. Positive β-amyloid PETs demonstrate increased radiotracer uptake throughout both the gray and white matter, and can be classified as moderately positive (BAPL 2) or diffusely positive (BAPL 3) based on qualitative evaluation of the intensity of β-amyloid-related uptake. For example, a BAPL 2 scan may show moderate uptake in just the frontal lobes with relatively subtle loss of gray-white matter differentiation (arrows), while a BAPL 3 scan may demonstrate intense uptake with clear loss of gray-white matter differentiation in multiple regions, including the bilateral frontal lobes and temporal lobes (arrows). Centiloid scores corresponding to each scan are also shown and illustrate that higher Centiloid values tend to occur with more qualitatively positive scans.

**Figure 4:**
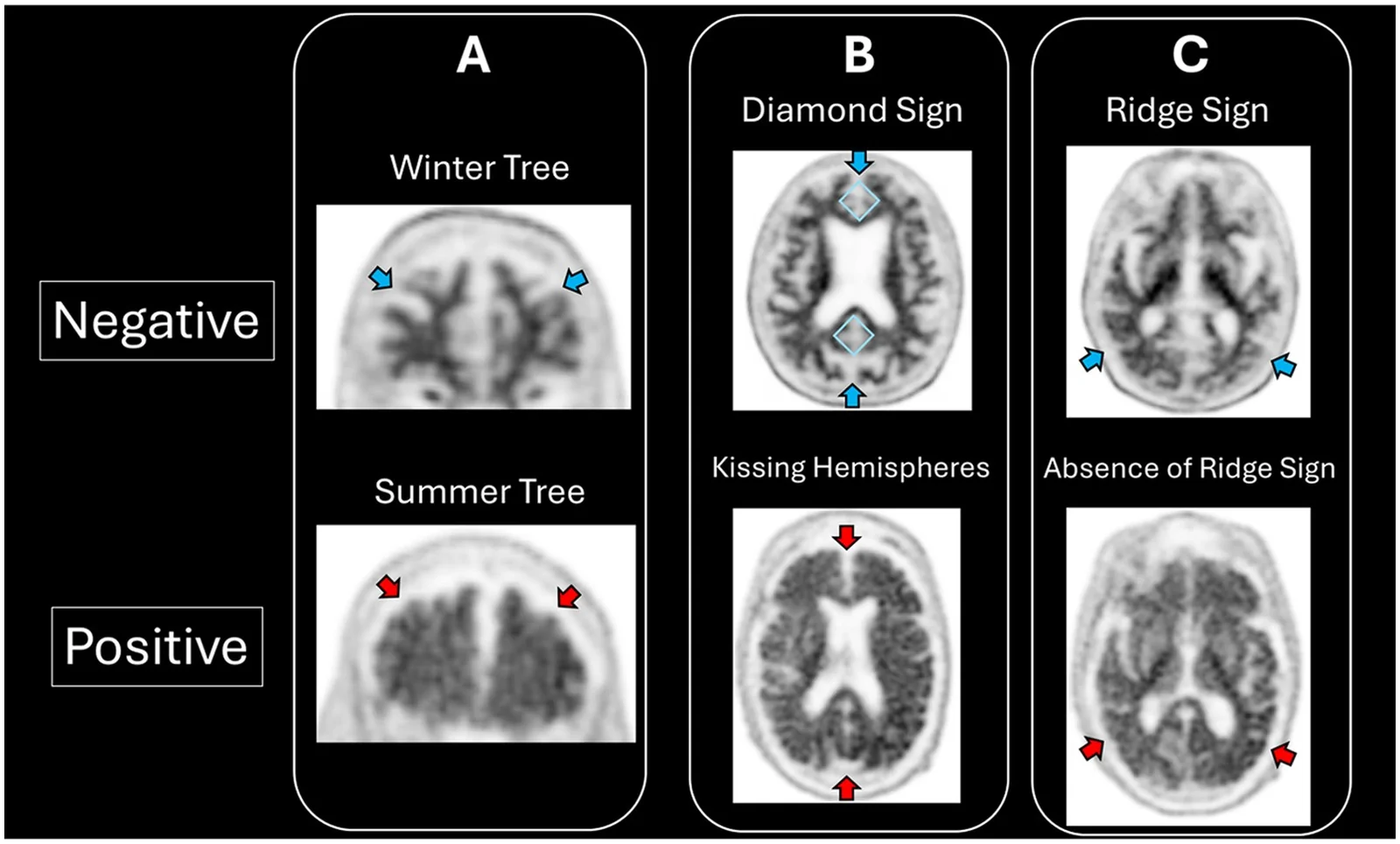
Examples of qualitative signs aiding interpretation of β-amyloid PET as negative or positive: representative cases performed with ^1^^8^F-florbetaben. Negative scans (top row) demonstrate only white-matter uptake, while positive scans (bottom row) additionally show gray matter uptake. These patterns of uptake result in recognizable signs. (A) The “Winter Tree” branching pattern on coronal slices highlights the appearance of white-matter uptake (blue arrows), while the “Summer Tree” pattern also shows gray matter uptake (red arrows), simulating the “fuller” appearance of a tree in summer. (B) The “Diamond Sign” can refer to a diamond-like appearance (blue arrows) in the interhemispheric regions on negative scans in the frontal lobes due to the lack of gray matter uptake; a similar appearance can be seen in the precuneus/posterior cingulate gyrus. Positive scans will have these regions “fill” with gray matter uptake, causing the “kissing hemispheres” sign (red arrows). (C) The presence of the “Ridge Sign” can be seen in negative scans of the lateral temporal regions, where the lack of gray matter uptake creates an irregular, ridge-like appearance (blue arrows). Positive scans will demonstrate a decrease in conspicuity of this ridge-like appearance (red arrows).

**Figure 5:**
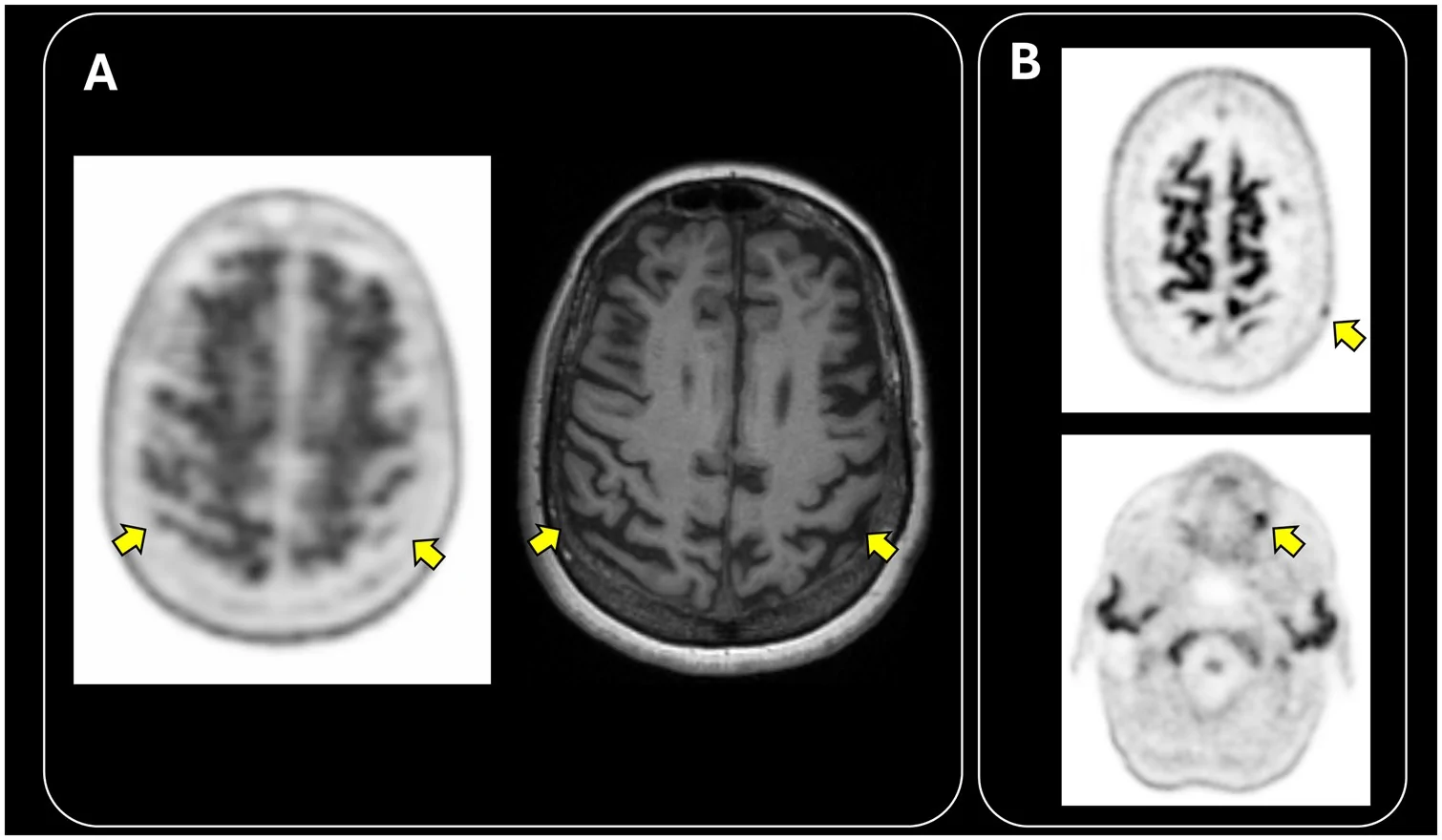
Examples of challenges in the qualitative interpretation of β-amyloid PET. (A) Example of parenchymal volume loss causing the qualitative appearance of a positive study to mimic a negative scan; the pattern of uptake in the parietal lobes on PET (left) does not appear to extend to the cortex (arrows), but this corresponds to areas of parenchymal volume loss on a T1-weighted MRI obtained within 1 month of the PET (right). In addition, the Centiloid value can provide aid in deciding on the positivity of a scan with volume loss: the Centiloid value in this case was 96.21. (B) The top image demonstrates an example of off-target focal radiotracer uptake in the left posterior scalp (arrow), of indeterminate significance and without soft tissue correlate on CT. The bottom image demonstrates focal off-target uptake in the left maxillary region (arrow).

The Centiloid score is a quantitative scale that can complement qualitative reads of amyloid brain PETs. The scale is based on studies of ^11^C-PiB binding in cognitively normal individuals and individuals with AD,^25^ with a score of 0 representing the average radiotracer uptake seen in cognitively normal individuals and a score of 100 representing the radiotracer uptake measured in individuals with AD. Therefore, an individual’s Centiloid score can be less than 0 or more than 100, depending on the uptake they exhibit relative to these populations.^25^ Equations are used to convert the standardized uptake value ratio (SUVR) (conventionally utilizing a cerebellar-reference region) from other amyloid tracers to the Centiloid scale, allowing translation between different clinically-approved tracers. While no formal guidelines on score interpretation exist, one approximate practice is that a score <10 is considered negative, >30 is positive, and the 10-30 range is an intermediate “gray zone”.^30^^,^^31^ However, although Centiloid scores are helpful supplements, qualitative interpretations supersede them. Several situations warrant caution when interpreting Centiloid scores, including elevated cerebellar-reference uptake (Figure 6A), motion artifact (Figure 6B), and focal uptake localized to a single anatomical region that may be outside the regions of interest for Centiloid quantification (Figure 6C).

**Figure 6:**
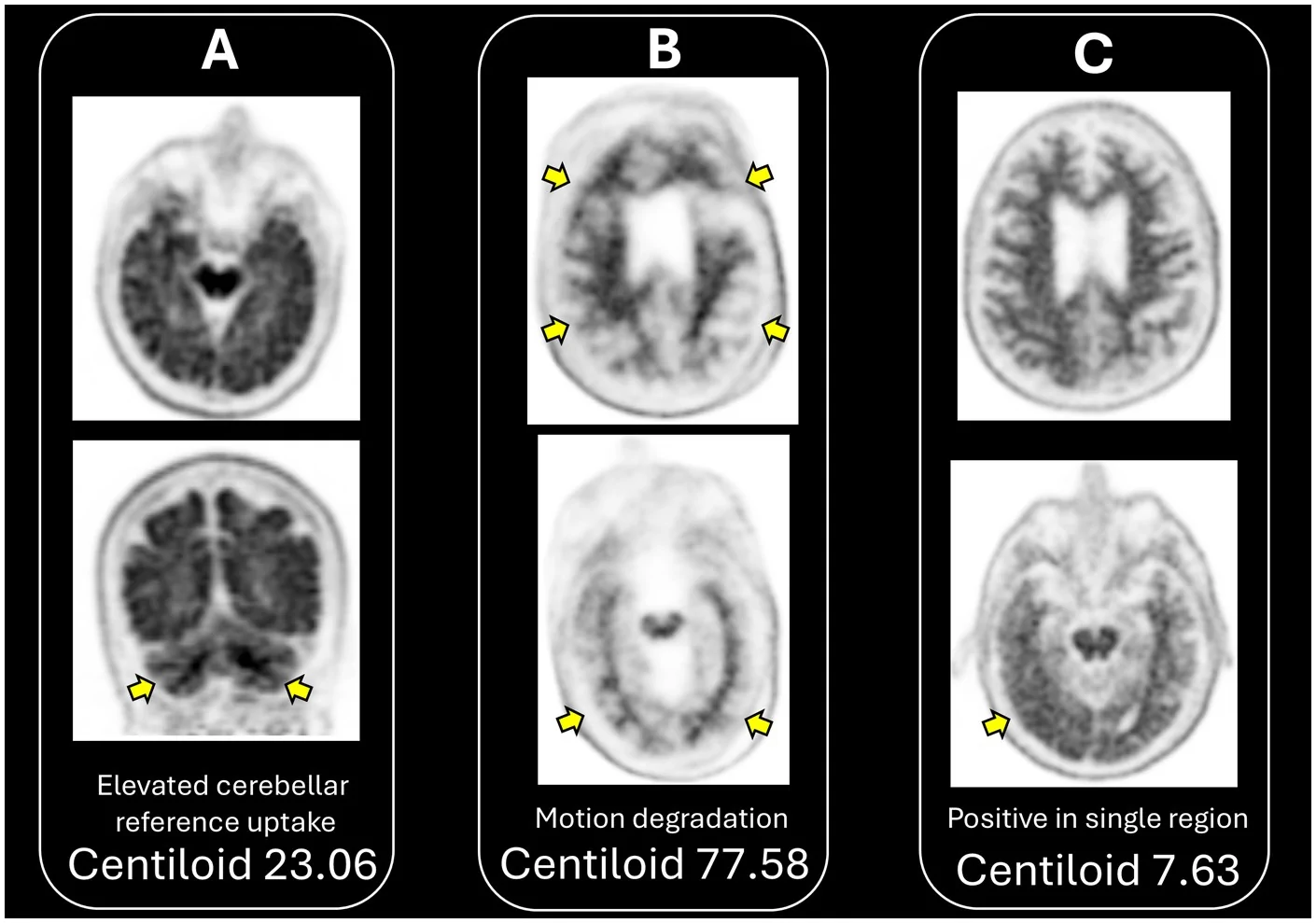
Examples of discordance between qualitative interpretation and Centiloid values in ^18^F-florbetaben brain PET. (A) Example of elevated cerebellar uptake (arrows) resulting in low Centiloid value for a qualitatively positive scan. (B) Example of motion artifact resulting in blurring of gray-white-matter differentiation throughout the brain (arrows) and artificially high Centiloid in a nondiagnostic scan. (C) Qualitatively positive scan due to uptake predominantly in the right temporal lobe (arrow) with a low (<10) Centiloid value that is typically seen with negative exams.

Amyloid PET is a routine part of clinical practice in the evaluation of dementia. It has demonstrated value in clinical management, with 1 study involving over 11 000 patients demonstrating that amyloid PET results changed clinical management in over 60% of patients.^32^ Amyloid PET can stratify subjects, as patients with positive amyloid scans have been reported to experience more severe cognitive decline compared to those with negative scans.^33^ Additionally, the Centiloid score has served an increasingly important clinical role with the growing use of amyloid-directed therapies, as it can track treatment effect magnitudes (Figure 7). While amyloid PET is a powerful diagnostic tool for AD, it is important to note that clinical context is necessary for its interpretation. Patients primarily diagnosed with non-AD pathologies, including PD and DLB, can demonstrate uptake on amyloid PET, and whether this represents co-occurrence of AD or incidental uptake should be carefully clinically considered as per the 2024 updates to the Alzheimer’s Association (AA) diagnostic framework.^18^ Finally, cognitively normal individuals can also have brain amyloid uptake. Therefore, amyloid PET imaging is generally reserved for patients with clinical suspicion for MCI or AD. Importantly, a negative scan makes AD highly unlikely as the primary cause of cognitive impairment. The AA and the Society of Nuclear Medicine and Molecular Imaging (SNMMI) published appropriate use criteria (AUC) designating amyloid PET as appropriate for patients with MCI or dementia when AD is suspected (particularly those <65, with atypical features, or when CSF biomarkers are equivocal) and for determining eligibility for amyloid-targeting therapies.^19^

**Figure 7:**
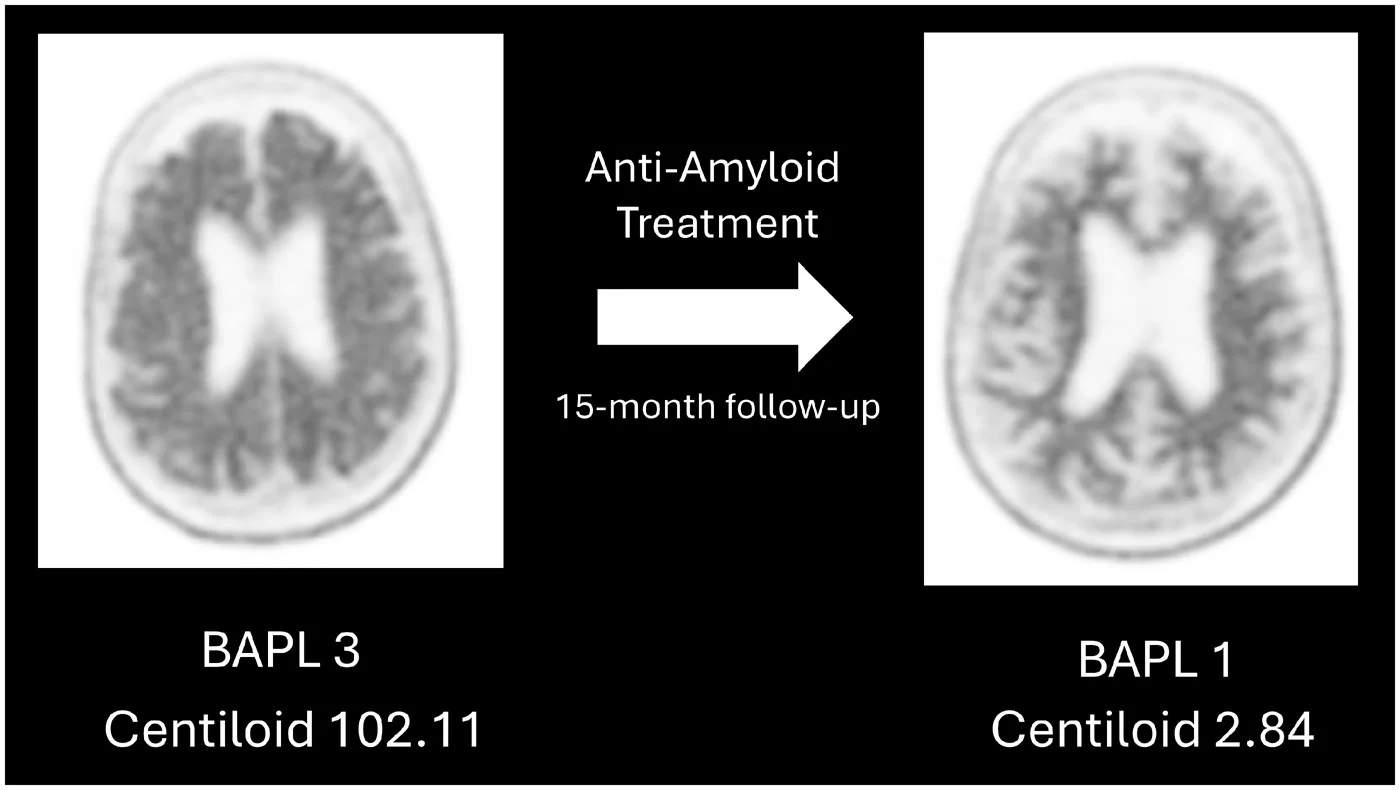
Example of reduction in cerebral β-amyloid burden following anti-amyloid treatment with the monoclonal antibody lecanemab. β-amyloid brain PETs utilizing ^18^F-florbetaben demonstrate a significant reduction in gray matter uptake 15 months after initiating anti-amyloid treatment.

## Tau

Tau is an intracellular cytoskeleton-associated protein that, when hyperphosphorylated, aggregates to form neurofibrillary tangles in AD. The spread of tau propagates along a stereotypic spatial trajectory summarized by the 7-stage Braak pathological classification, beginning in the brainstem nuclei (stage 0) and spreading to the hippocampus and medial temporal lobe (MTL; stage I-II), then the lateral temporal lobe (stage III-IV), and finally to the rest of the neocortex (stage V-VI), with the cerebellum being one of the last affected areas.^30^^,^^34^ Patient symptoms tend to correlate better with location of tau deposition than amyloid.^30^

Tau PET is emerging as an important tool in dementia neuroimaging.^30^ Tau PET provides an opportunity to assess *in vivo* the regional distribution of tau protein aggregate deposition. Therefore, tau PET has great potential prognostic value in AD and can indicate where a patient is in the course of disease at the time of the scan. Thus, tau PET is well-suited to complement β-amyloid PET: while β-amyloid PET can suggest an AD diagnosis in the correct clinical setting, tau PET can assess the spread of neuropathological changes in the brain.

In AD, tau PET typically demonstrates uptake in the cortex (Figure 8). According to the AA/SNMMI AUC, positive scans demonstrate increased cortical uptake in the posterolateral temporal, occipital, or parietal/precuneus regions, and may also demonstrate frontal uptake.^19^^,^^30^ The inferior cerebellum serves as the reference standard to compare uptake due to its late involvement in AD. There are 2 current FDA-approved radiotracers: ^18^F-flortaucipir and ^18^F-MK-6240 (^18^F-florquinitau)^35^^,^^36^ (Table 2).

**Figure 8:**
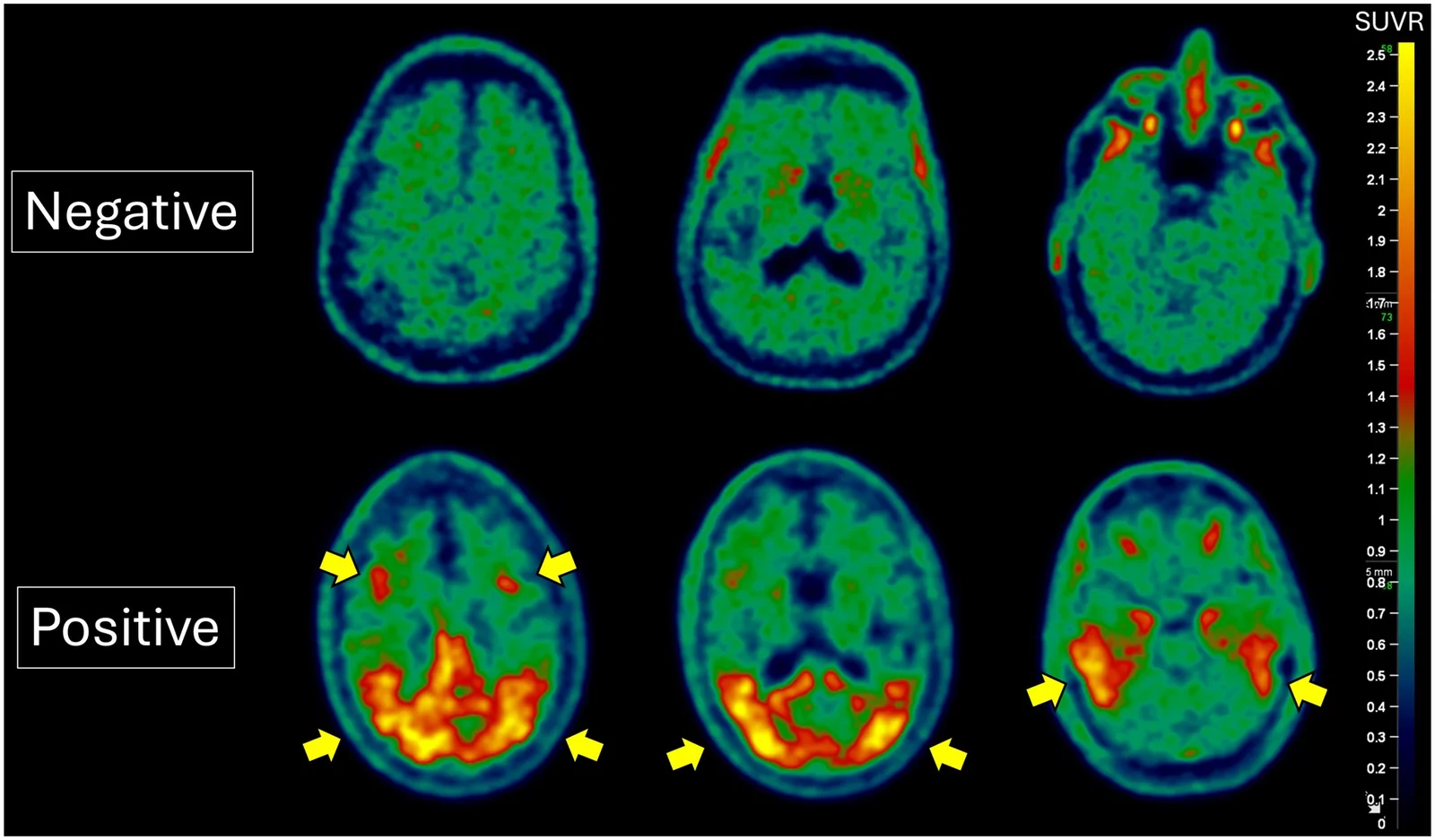
Negative and positive tau PETs using ^18^F-flortaucipir on representative axial slices in 2 different patients, measured by cerebellar-reference SUVR. Negative tau PET demonstrates limited radiotracer uptake throughout the brain. Positive ^18^F-flortaucipir PET demonstrates increased uptake in characteristic regions, including in the posterolateral temporal lobes and parietal lobes (arrows), as well as a smaller amount of uptake in the bilateral frontal lobes.

^18^F-flortaucipir has high sensitivity and specificity for distinguishing AD from other neurodegenerative dementias.^35^ However, ^18^F-flortaucipir uptake in the MTL may not be a reliable marker of AD, as this region is susceptible to age-related tau accumulation and off-target binding to the choroid plexus. Additionally, ^18^F-flortaucipir exhibits off-target binding to monoamine oxidase (MAO) in the basal ganglia and choroid plexus, as well as nonspecific uptake in the white matter.^30^

A newer recently FDA-approved second-generation tracer, ^18^F-MK-6240, has been found to have higher binding affinity for tau but demonstrates off-target uptake to multiple locations, including the substantia nigra, globus pallidus, pineal gland, and possibly the meninges.^37–39^  ^18^F-MK-6240 has been shown to have reduced background signal and higher tau positivity in the MTL at lower Aβ burden, allowing for earlier detection of disease (especially in Braak stages I-III where ^18^F-flortaucipir has limited sensitivity). Note that caution must be taken when comparing studies with ^18^F-flortaucipir and ^18^F-MK-6240 given their different binding characteristics.

No standardized quantitative metric for tau PET is currently applicable across radiotracers, as the Centiloid score is in β-amyloid PET. One area of research is the CenTauR framework, which can account for the binding magnitudes of different radiotracers within predefined anatomic regions to provide a quantitative measure of cerebral tau burden.^40^ Since the spatial distribution of tau deposition is also important for AD prognostication, methods of quantitatively reporting the extent of tau distribution are also being investigated.^41^ Finally, AD therapies targeting tau deposition are under active development, and standardizing quantitative metrics to follow the effects of these potential therapies will be necessary.^30^

Non-AD tauopathies are defined by tau protein aggregates that are structurally different from the neurofibrillary tangles associated with AD; nevertheless, neurofibrillary tangles can also be present in non-AD tauopathies.^42^ Examples of non-AD tauopathies include FTLD-tau, which can underlie clinical syndromes such as behavioral-variant frontotemporal dementia (bvFTD), as well as CBD and PSP.^14^  ^18^F-Flortaucipir can demonstrate uptake in these conditions, as well as in PD; however, radiotracer binding is generally poor and does not closely associate with neuropathological evidence of these diseases.^43^^,^^44^ Current tau tracers may also bind to α-synuclein or TDP-43, reducing the specificity of tau PET.^44^ Tau protein isoforms are subcategorized into 3R or 4R forms depending on the number of repeats of the tau microtubule binding domain. Different tauopathies have different relative ratios of 3R and 4R tau protein isoforms, with Parkinson-plus syndromes predominantly forming 4R-based aggregates in microglia.^44^  ^18^F-flortaucipir primarily targets mixed 3R/4R tau isoforms seen in neurofibrillary tangles and has lower affinity for pure 3R or 4R tauopathies,^45^ so targeting specific tau isoforms may form a basis for the development of new, more specific radiotracers. The AA/SNMMI AUC deems tau PET as appropriate in patients with suspected AD who are aged <65 years or have atypical clinical features, and for informing prognosis and treatment eligibility.^34^ The combination of tau and amyloid tracers together has not been extensively studied, but could be used to further the diagnosis and treatment of AD and other neurodegenerative diseases.

## Emerging radiotracers: TSPO and other neuroinflammation tracers

Inflammation plays a central role in the pathogenesis of numerous neurodegenerative processes, including AD, PDD, FTLD, Huntington’s disease (HD), and amyotrophic lateral sclerosis (ALS). Translocator protein (TSPO), an outer mitochondrial membrane protein, is the most widely studied PET target for neuroinflammation.^46^ It is expressed at low levels in the normal human brain, but is upregulated with brain injury and inflammation due to an increase in activated microglia and reactive astrocytes.^47^

TSPO PET has demonstrated the most consistent findings in AD, where it is postulated to be increased in plaque-associated phagocytic microglia. Meta-analyses reveal increases in TSPO signal in cortical gray matter and cortico-limbic regions of AD patients compared to healthy controls.^48^ Importantly, TSPO upregulation occurs early in disease progression,^49^ though it more closely follows tau patterns than amyloid patterns.^48^

Although findings have been less consistent, TSPO elevation has been documented in other neurodegenerative diseases. In PDD, TSPO elevations have been shown in prodromal stages, likely due to microglial activation. Variable findings have also been seen in FTLD, HD and ALS.^46^

While there are no FDA-approved TSPO tracers, ^11^C-PK11195 was the first tracer to show promise in *in vivo* studies and is used as a research tracer in humans.^48^ TSPO has potential for drug development, particularly for identifying patients likely to respond to anti-inflammatory treatments. However, TSPO-targeted imaging poses several limitations, including a lack of cellular specificity, the inability to differentiate protective glial proliferation from neurotoxic activation states, and high- versus low-affinity binding variations caused by the rs6971 genetic polymorphism.^46^

Other neuroinflammation tracers are in various stages of development, including those specific to microglia (CSF1R, P2X7R, CB2R), astrocytes (MAO-B, I2BS), myeloperoxidase activity (^18^F-MAPP),^50^ and cyclooxygenase (COX-1 and COX-2).^48^ However, none have achieved routine clinical use.

## Emerging radiotracers: synaptic density tracers

Synaptic loss has been postulated to be involved in various neurodegenerative processes.^51^ While traditionally only quantifiable through tissue sampling, radiotracers targeting synaptic vesicle glycoprotein 2A (SV2A), a transmembrane protein expressed in synaptic vesicles, enable noninvasive quantification of synaptic density. SV2A is found throughout all gray matter structures, whereas the related protein, SV2B, is expressed only in subcortical areas. The structures of these tracers are based on the antiepileptic drug levetiracetam. The most widely studied of these tracers is ^11^C-UCB-J (Table 3), which demonstrated excellent kinetic properties, high gray matter uptake, and reproducibility.^52^ However, due to the short 20-min half-life of carbon-11 and inherently long scan times, ^18^F-labeled tracers (^18^F-SynVesT-1 and ^18^F-SynVesT-2) were developed. ^18^F-SynVest-2 demonstrates highly specific brain uptake and fast brain kinetics.^53^ The centrum semiovale and, more recently, the cerebellum have been suggested as reference regions for quantification due to their low SV2A expression.^51^

**Table 3:** Examples of next-generation radiotracers in the evaluation of neurodegeneration.

Next-generation radiotracer	Target	Off-target binding
**^18^F-ACI-12589 (PD)**	α-Synuclein	Tau and beta amyloid
**^11^C-PK11195**	TSPO (primarily in microglia and astrocytes)	Vascular endothelium, plasma proteins, choroid plexus, ependymal cells
**^11^C-UCB-J**	SV2A	Phosphorylated tau and white matter

Abbreviations: PD = Parkinson’s disease, SV2A = synaptic vesicle glycoprotein 2A, TSPO = translocator protein.

Promising results have been observed across several diseases. AD has been the most widely investigated, with studies showing decreased ^11^C-UCB-J uptake in the MTL, hippocampus, amygdala, thalamus, and neocortex. These changes are less pronounced than with FDG-PET, except in the MTLs where they are comparable.^54^ Decreased SV2A uptake inversely correlates with tau and amyloid burden, though synaptic loss parallels tau pathology more closely than amyloid.^55^ In PD, reduced SV2A uptake is most notable in the caudate, thalamus, substantia nigra, red nucleus, and locus coeruleus.^51^ In DLB, the findings were less conclusive, with decreased uptake in multiple cortical regions, most notably the insular cortex, and decreased FDG uptake in the occipital lobes being much more pronounced than decreases in SV2A uptake.^56^ In FTLD, ^11^C-UCB-J uptake is reduced in the frontotemporal regions.^57^ Correlations between synaptic density and clinical manifestations have been reported across several studies in AD, PD, DLB, FTLD, PSP, CBD, and HD.^51^

## Dopaminergic radiotracers: ^18^F-DOPA and α-synuclein

Fluorodopa F-18 (^18^F-DOPA) and α-synuclein radiotracers target distinct pathophysiologic processes and provide complementary approaches to neuroimaging of Parkinsonian syndromes. ^18^F-DOPA is taken up by presynaptic dopaminergic nerve terminals in the striatum.^58^ Decreased striatal uptake is seen among the synucleinopathies, including PD, DLB, and multiple system atrophy (MSA). Decreased uptake progresses with a posterior-to-anterior gradient, similar to ^123^I-ioflupane imaging, initially affecting the posterior putamen with relative sparing of the caudate head (Figure 9). This creates the characteristic “dot” sign, compared with the “comma” sign in healthy controls. While ^18^F-DOPA cannot distinguish between specific parkinsonian syndromes or directly visualize the underlying protein aggregates, it has a high sensitivity and specificity for neurodegenerative Parkinsonism (Table 2).^59^ Although ^18^F-DOPA PET allows for high-resolution functional assessment of dopaminergic neurons, it has limited adoption in the United States, with centers tending to use the cost-effective alternative ^123^I-ioflupane DaTscan single-photon emission computed tomography (SPECT).^60^

**Figure 9:**
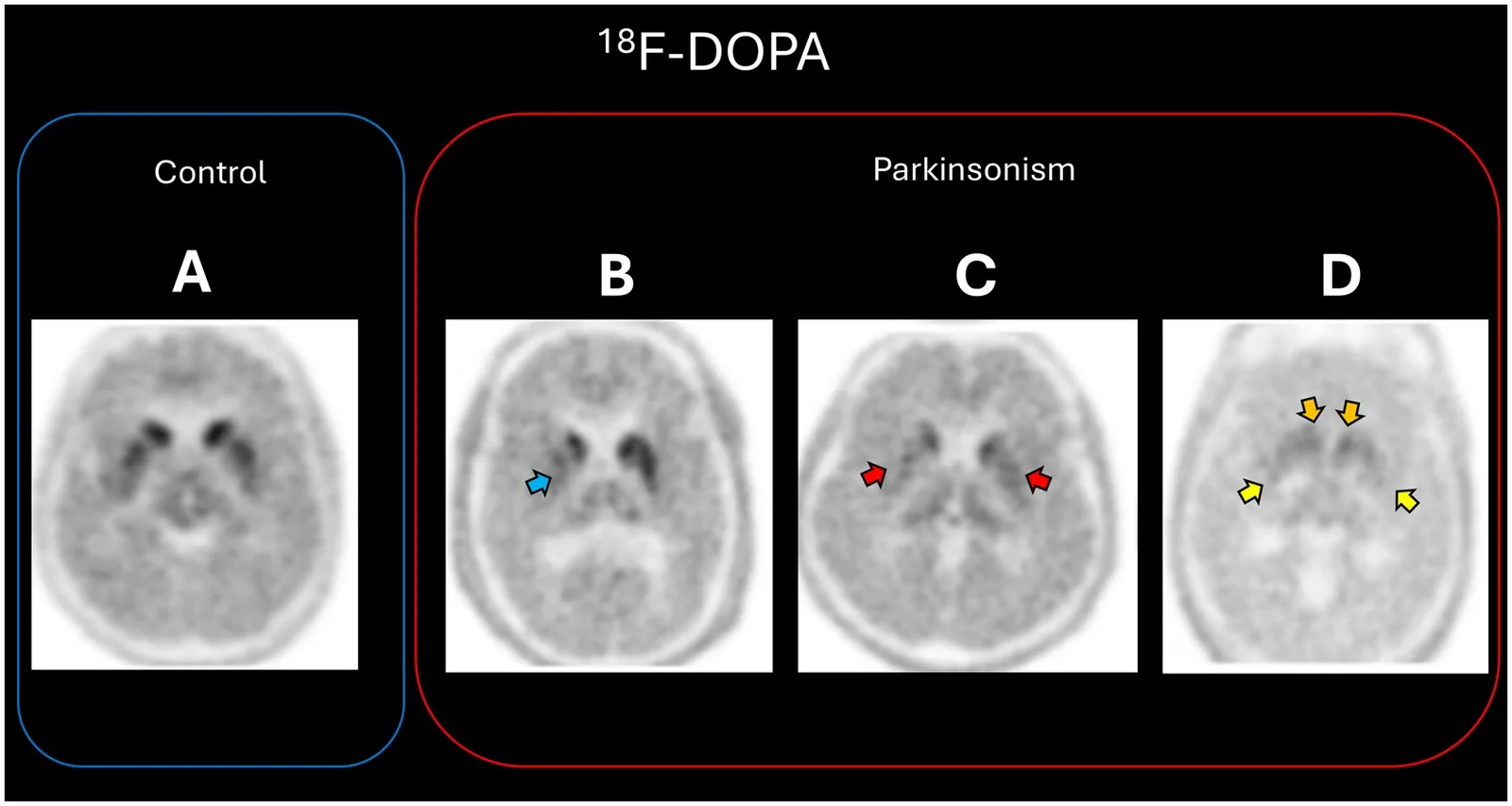
Control and positive examples of ^18^F-DOPA PET cases. Representative axial slices at the level of the striatum are shown. (A) Control patient with relatively symmetric uptake in the bilateral caudates and putamina. (B) Decreased uptake in the right putamen (blue arrow) but preserved uptake in the left putamen and bilateral caudate heads. (C) Decreased uptake in the bilateral putamina, right worse than left (red arrows), but with preserved uptake in the bilateral caudate heads. (D) Advanced case with decreased uptake in the bilateral putamina (yellow arrows), as well as the bilateral caudate heads (orange arrows).

Conversely, α-synuclein PET tracers directly target protein aggregates and show promise in differentiating specific synucleinopathies (such as MSA) from other neurodegenerative diseases. Preclinical and *ex vivo* studies provide evidence of target engagement with pathological α-synuclein inclusions: in PD and DLB, α-synuclein pathology is predominantly neuronal and occurs in Lewy bodies and neurites, whereas in MSA it is predominantly found in oligodendrocytes as glial cytoplasmic inclusions.^61^^,^^62^  ^18^F-ACI-12589 is currently one of the most studied of these tracers and demonstrates increased uptake in the middle cerebellar peduncles and cerebellar white matter in MSA-C (cerebellar phenotype) patients and the lentiform nuclei in MSA-P (Parkinsonian phenotype) patients.^61^ However, no specific regional pattern was seen in other neurodegenerative processes, including PD and DLB.^61^  ^18^F-C05-05 is another α-synuclein tracer that showed increased uptake in the midbrain of patients with PD and DLB compared to healthy controls.^63^ Limitations of α-synuclein PET include tracer-dependent off-target binding to tau, amyloid, and white-matter structures (Table 3), as well as variable affinity for distinct α-synuclein aggregate conformations across different synucleinopathies.^62^

## Potential for theranostics in neurodegenerative disease

Theranostics combines diagnostic imaging with targeted therapy, typically using agents that recognize the same molecular target.^64^ In neurodegenerative disease, amyloid and tau PET have enabled *in vivo* characterization of disease-specific pathology, while recent disease-modifying therapies for AD, including lecanemab and donanemab, have demonstrated the clinical potential of pathology-directed treatment. However, there are currently no FDA-approved radiotheranostic agents for neurodegenerative disorders.

Molecular PET nevertheless provides a potential foundation for neurotheranostics by identifying and quantifying pathological targets that could be exploited for targeted therapy. Radiolabeled therapeutic candidates can also be used to characterize biodistribution, pharmacokinetics, and, where appropriate, target engagement. Importantly, PET localization does not necessarily predict the distribution or therapeutic efficacy of a corresponding therapeutic agent, particularly in the brain, where the blood–brain barrier (BBB) remains a major obstacle to drug delivery.^65^

Emerging BBB-transport technologies may help address this limitation. For example, the Brain Shuttle platform uses receptor-mediated transcytosis (specifically through transferrin receptor 1) to enhance delivery of biologics across the BBB; trontinemab, an anti-amyloid antibody incorporating this approach, has progressed into clinical development.^66^ Magnetic nanosystems are also promising as potential theranostic platforms since magnetic nanoparticles can serve as contrast agents for MRI and as drug delivery systems.^67^ Together, advances in molecular PET, radiopharmaceutical development, and BBB-penetrating delivery strategies may establish a future neurotheranostic paradigm in which pathological targets are identified, therapeutics are selectively delivered, and treatment response is quantitatively monitored.

## Current and future applications of PET in neurodegeneration

Clinical applications currently focus on several key molecular targets, as we have described. A combination of FDG, β-amyloid, and tau brain PET can help characterize a dementia diagnosis by elucidating metabolic abnormalities and pathological protein aggregation, thereby helping guide patients toward the appropriate treatment pathway. Future PET-based diagnostic tools in dementia may be able to clinically image neuroinflammation and synaptic density through engagement of additional molecular targets. Similarly, in Parkinsonism, existing dopaminergic imaging approaches, including ^18^F-DOPA PET and ^123^I-ioflupane (DaTscan) SPECT, can detect presynaptic dopaminergic deficits. These approaches may eventually be complemented by investigational PET tracers targeting α-synuclein aggregates, potentially providing more disease-specific molecular information.

Once structural pathologies are excluded using brain MRI, patients presenting with clinical symptoms of neurodegeneration can be further evaluated using brain PET, potentially utilizing several different radiotracers depending on clinical goals (Figure 10). Suspected AD can be evaluated with an amyloid PET scan, which, if positive, can subsequently lead to a tau PET to provide prognostic information. In patients with a negative amyloid PET scan, an FDG-PET scan can help identify characteristic hypometabolic patterns associated with non-AD conditions and clarify the underlying diagnosis. On the other hand, a patient with movement symptoms or clinical concern for Parkinsonism could first undergo a DaTscan/^18^F-DOPA scan. If the DaTscan/^18^F-DOPA scan is negative, FDG-PET can again be used to identify areas of regional hypometabolism. In the future, even if a patient has negative amyloid PET and FDG-PET scans and does not yet have a clear neurodegenerative disease diagnosis, other currently investigational tracers targeting additional aspects of neurodegenerative disease pathophysiology may become available for clinical use, potentially enabling further diagnostic evaluation.

**Figure 10:**
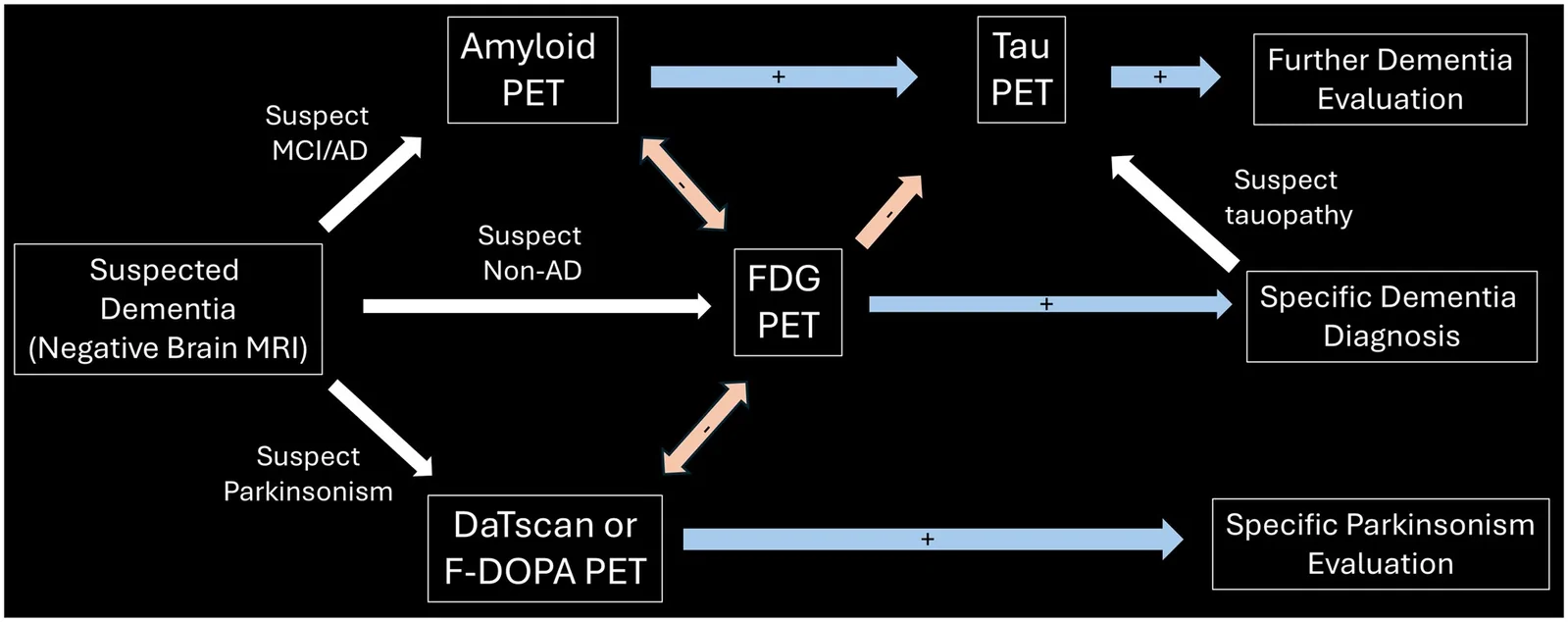
PET-based example diagnostic algorithm for patients with suspected dementia in which structural causes have been excluded with brain MRI. Blue arrows represent positive results, orange arrows represent negative or inconclusive results, and white arrows represent imaging selection based on the predominant clinical suspicion. The initial imaging study may be selected according to the predominant clinical suspicion: β-amyloid PET for suspected mild cognitive impairment (MCI) or Alzheimer disease (AD), FDG-PET for suspected non-AD dementia, or DaTscan/^18^F-DOPA PET for suspected Parkinsonism. A negative β-amyloid PET can be followed with an FDG-PET, and vice versa, as can a negative DaTscan/^18^F-DOPA PET (double-headed arrows). A positive β-amyloid PET may be followed by tau PET for disease staging and prognostic assessment when clinically appropriate. A positive DaTscan/^18^F-DOPA PET can supplement clinical evaluation of Parkinsonism. FDG-PET may support a specific dementia diagnosis based on characteristic patterns of hypometabolism but may also be inconclusive, requiring integration with other clinical, imaging, and biomarker findings.

## Challenges in translation of radiotracers from preclinical to clinical

Multiple challenges exist in the clinical translation of radiotracers for neurodegeneration, including developing tracers that can cross the BBB and possess adequate pharmacokinetics to allow for imaging.^68^ Radiotracers need to be small and lipophilic to cross the BBB without having affinity for efflux pumps that can immediately pump them back out of the brain tissue.^68^ After crossing the BBB, specific pharmacokinetics are required; the tracer needs to bind to the target rapidly while clearing from non-target tissue before the radioisotope decays. This is especially challenging when targeting early stages of neurodegeneration, where concentrations of abnormal protein targets are low. Additionally, structural heterogeneity of protein aggregates, for example, 3R, 4R, and mixed 3R/4R isoforms of tau, create distinct binding pocket geometries that prevent a single tracer from binding all pathological forms.^44^ Although no animal model can completely recapitulate human diseases, well-characterized animal models for several dementias provide a valuable platform for preclinical radiotracer evaluation, including characterization of *in vivo* pharmacokinetics and pharmacodynamics, biodistribution, disease-associated signal changes, and correlation of PET signal with target expression and pathology.^69^ Beyond biological constraints, translating these tracers into clinical use requires a rigorous FDA approval involving extensive preclinical safety validation in healthy rodents at high escalating doses, radiation dosimetry, and manufacturing standards.^70^

## Summary and conclusions

PET-based molecular neuroimaging is a rapidly growing field with tremendous potential to improve human health, especially given the increasing prevalence of neurodegenerative diseases. Many tracers are now in routine clinical use, and emerging tracers show great promise for improving diagnosis, prognosis, and treatment monitoring in neurodegenerative diseases. These advancements encompass diverse biological targets, ranging from established metabolic markers to novel protein-specific pathways.

## Supplementary Material

umag046_Supplementary_Data

## References

[1] 2024 Alzheimer’s disease facts and figures. Alzheimers Dement. 2024;20(5):3708-3821. 10.1002/alz.13809 PMC11095490 38689398

[2] Borghammer P , Okkels N, Weintraub D. Parkinson’s disease and dementia with Lewy bodies: one and the same. J Park Dis. 2024;14(3):383-397. 10.3233/JPD-240002 PMC11091584 38640172

[3] Wilkie RZ , Ho JY. Life expectancy and geographic variation in mortality: an observational comparison study of six high-income Anglophone countries. BMJ Open. 2024;14(7):e079365. 10.1136/bmjopen-2023-079365 PMC11407213 39138004

[4] Liss JL , Seleri Assuncao S, Cummings J, et al Practical recommendations for timely, accurate diagnosis of symptomatic Alzheimer’s disease (MCI and dementia) in primary care: a review and synthesis. J Intern Med. 2021;290(2):310-334. 10.1111/joim.13244 PMC8359937 33458891

[5] Foltynie T , Bruno V, Fox S, Kühn AA, Lindop F, Lees AJ. Medical, surgical, and physical treatments for Parkinson’s disease. Lancet. 2024;403(10423):305-324. 10.1016/S0140-6736(23)01429-0 38245250

[6] van Dyck CH , Swanson CJ, Aisen P, et al Lecanemab in early Alzheimer’s disease. N Engl J Med. 2023;388(1):9-21. 10.1056/NEJMoa2212948 36449413

[7] Sims JR , Zimmer JA, Evans CD, et al Donanemab in early symptomatic Alzheimer disease: the TRAILBLAZER-ALZ 2 randomized clinical trial. JAMA. 2023;330(6):512-527. 10.1001/jama.2023.13239 PMC10352931 37459141

[8] Spano M , Roytman M, Aboian M, Saboury B, Franceschi A, Chiang GC. Brain PET imaging: approach to cognitive impairment and dementia. PET Clin. 2023;18(1):103-113. 10.1016/j.cpet.2022.09.006 PMC9713600 36442959

[9] Jack CR , Bennett DA, Blennow K, et al NIA-AA research framework: toward a biological definition of Alzheimer’s disease. Alzheimers Dement. 2018;14(4):535-562. 10.1016/j.jalz.2018.02.018 PMC5958625 29653606

[10] Gadhave DG , Sugandhi VV, Jha SK, et al Neurodegenerative disorders: mechanisms of degeneration and therapeutic approaches with their clinical relevance. Ageing Res Rev. 2024;99:102357. 10.1016/j.arr.2024.102357 38830548

[11] Stefanis L. α-Synuclein in Parkinson’s disease. Cold Spring Harb Perspect Med. 2012;2(2):a009399. 10.1101/cshperspect.a009399 PMC3281589 22355802

[12] Higuchi M , Tagai K, Takahata K, Endo H. Advances in PET imaging of protein aggregates associated with neurodegenerative disease. Nat Rev Neurol. 2025;21(9):506-522. 10.1038/s41582-025-01126-2 40790282

[13] Klunk WE , Engler H, Nordberg A, et al Imaging brain amyloid in Alzheimer’s disease with Pittsburgh compound-B. Ann Neurol. 2004;55(3):306-319. 10.1002/ana.20009 14991808

[14] Anzai Y , Minoshima S. The role of brain MR and FDG-PET in the diagnosis of neurodegenerative disease. Eur Radiol. 2026;36(3):1746-1757. 10.1007/s00330-025-11940-3 PMC12963154 40903623

[15] Nerattini M , Abenavoli EM, Berti V. PET/CT in movement disorders: update. Semin Nucl Med. 2025;55(4):565-576. 10.1053/j.semnuclmed.2025.03.007 40320359

[16] Burkett BJ , Johnson DR, Lowe VJ. Evaluation of neurodegenerative disorders with amyloid-β, tau, and dopaminergic PET imaging: interpretation pitfalls. J Nucl Med Off Publ Soc Nucl Med. 2024;65(6):829-837. 10.2967/jnumed.123.266463 PMC13671385 38664015

[17] Soderlund KA , Austin MJ, et al; Expert Panel on Neurological Imaging. ACR appropriateness criteria® dementia: 2024 update. J Am Coll Radiol JACR. 2025;22(5S):S202-S233. 10.1016/j.jacr.2025.02.031 40409878

[18] Jack CR , Andrews JS, Beach TG, et al Revised criteria for diagnosis and staging of Alzheimer’s disease: Alzheimer’s Association Workgroup. Alzheimers Dement J Alzheimers Assoc. 2024;20(8):5143-5169. 10.1002/alz.13859 PMC11350039 38934362

[19] Rabinovici GD , Knopman DS, Arbizu J, et al Updated appropriate use criteria for amyloid and tau PET: a report from the Alzheimer’s Association and Society for Nuclear Medicine and Molecular Imaging Workgroup. J Nucl Med. 2025;66(Suppl 2):S5-S31. 10.2967/jnumed.124.268756 PMC12794165 39778970

[20] Clark CM , Pontecorvo MJ, Beach TG, et al Cerebral PET with florbetapir compared with neuropathology at autopsy for detection of neuritic amyloid-β plaques: a prospective cohort study. Lancet Neurol. 2012;11(8):669-678. 10.1016/S1474-4422(12)70142-4 22749065

[21] Curtis C , Gamez JE, Singh U, et al Phase 3 trial of flutemetamol labeled with radioactive fluorine 18 imaging and neuritic plaque density. JAMA Neurol. 2015;72(3):287-294. 10.1001/jamaneurol.2014.4144 25622185

[22] Sabri O , Sabbagh MN, Seibyl J, et al Florbetaben PET imaging to detect amyloid beta plaques in Alzheimer’s disease: phase 3 study. Alzheimers Dement J Alzheimers Assoc. 2015;11(8):964-974. 10.1016/j.jalz.2015.02.004 25824567

[23] Landau SM , Thomas BA, Thurfjell L, et al Amyloid PET imaging in Alzheimer’s disease: a comparison of three radiotracers. Eur J Nucl Med Mol Imaging. 2014;41(7):1398-1407. 10.1007/s00259-014-2753-3 PMC4055504 24647577

[24] Cho SH , Choe YS, Kim YJ, et al Head-to-head comparison of ^18^F-florbetaben and ^18^F-flutemetamol in the cortical and striatal regions. J Alzheimers Dis JAD. 2020;76(1):281-290. 10.3233/JAD-200079 PMC9711935 32474468

[25] Iaccarino L , Burnham SC, Tunali I, et al A practical overview of the use of amyloid-PET Centiloid values in clinical trials and research. NeuroImage Clin. 2025;46:103765. 10.1016/j.nicl.2025.103765 PMC11960669 40101674

[26] Sabri O , Seibyl J, Rowe C, Barthel H. Beta-amyloid imaging with florbetaben. Clin Transl Imaging. 2015;3(1):13-26. 10.1007/s40336-015-0102-6 PMC4339690 25741488

[27] Lundeen TF , Seibyl JP, Covington MF, Eshghi N, Kuo PH. Signs and artifacts in amyloid PET. Radiogr Rev Publ Radiol Soc N Am Inc. 2018;38(7):2123-2133. 10.1148/rg.2018180160 30422768

[28] Piramal Imaging Limited. Neuraceq Summary of Product Characteristics. Accessed April 14, 2026. https://neuraceq.com/hcp/non-us/Appendix%201%20DOC_950%20v11.0.pdf

[29] Skyles T , Bouchal SM, Giarratana A, et al PET imaging in Alzheimer disease in the era of antiamyloid therapy in the United States: clinical utility, quantification, and policy landscape. J Nucl Med Technol. 2026;54(1):10-17. 10.2967/jnmt.125.271835 PMC12963684 41667280

[30] Chiotis K , Wang Y, La Joie R, Rabinovici GD. The role of amyloid-β and tau PET in the new era of Alzheimer disease therapies. J Nucl Med Off Publ Soc Nucl Med. 2026;67(2):175-185. 10.2967/jnumed.124.268339 PMC13671013 41290365

[31] Blazhenets G , Soleimani-Meigooni DN, Chiotis K, et al Amyloid PET quantitation and Centiloid thresholds in the diagnosis of Alzheimer disease: an individual participant data meta-analysis. JAMA. 2026;336(13):1135-1148. 10.1001/jama.2026.13116 PMC13366243 42441390

[32] Rabinovici GD , Gatsonis C, Apgar C, et al Association of amyloid positron emission tomography with subsequent change in clinical management among medicare beneficiaries with mild cognitive impairment or dementia. JAMA. 2019;321(13):1286-1294. 10.1001/jama.2019.2000 PMC6450276 30938796

[33] Doraiswamy PM , Sperling RA, Johnson K, et al Florbetapir F 18 amyloid PET and 36-month cognitive decline: a prospective multicenter study. Mol Psychiatry. 2014;19(9):1044-1051. 10.1038/mp.2014.9 PMC4195975 24614494

[34] Braak H , Del Tredici K. The preclinical phase of the pathological process underlying sporadic Alzheimer’s disease. Brain J Neurol. 2015;138(Pt 10):2814-2833. 10.1093/brain/awv236 26283673

[35] Ossenkoppele R , Rabinovici GD, Smith R, et al Discriminative accuracy of [18F]flortaucipir positron emission tomography for Alzheimer disease vs other neurodegenerative disorders. JAMA. 2018;320(11):1151-1162. 10.1001/jama.2018.12917 PMC6233630 30326496

[36] Kinarney M , Downs M. Lantheus announces FDA approval of TAUKLARIFY^TM^ (florquinitau F 18 injection), an F18-labeled tau PET imaging agent for Alzheimer’s disease. Lantheus Holdings. 2026. Accessed August 20, 2026. https://lantheusholdings.gcs-web.com/news-releases/news-release-details/lantheus-announces-fda-approval-tauklarifytm-florquinitau-f-18

[37] Povala G , Bellaver B, Lussier FZ, et al Comparison of [18F]flortaucipir and [18F]MK6240 for the detection of tau pathology in Alzheimer’s disease (HEAD): a multicentre, prospective, cross-sectional, within-participant study. Lancet. 2026;407(10544):2217-2226. 10.1016/S0140-6736(26)00417-4 PMC13656316 42208563

[38] Leuzy A , Pascoal TA, Strandberg O, et al A multicenter comparison of [18F]flortaucipir, [18F]RO948, and [18F]MK6240 tau PET tracers to detect a common target ROI for differential diagnosis. Eur J Nucl Med Mol Imaging. 2021;48(7):2295-2305. 10.1007/s00259-021-05401-4 PMC8175317 34041562

[39] Ishii K , Yamada T, Hanaoka K, et al Off-target binding of ^18^F-MK-6240 tau PET imaging demonstrated by high spatial resolution dedicated brain PET scanner. Ann Nucl Med. 2026;40(5):597-604. 10.1007/s12149-026-02161-9 PMC13124815 41627725

[40] Leuzy A , Raket LL, Villemagne VL, et al Harmonizing tau positron emission tomography in Alzheimer’s disease: the CenTauR scale and the joint propagation model. Alzheimers Dement J Alzheimers Assoc. 2024;20(9):5833-5848. 10.1002/alz.13908 PMC11497758 39041435

[41] Therriault J , Pascoal TA, Lussier FZ, et al Biomarker modeling of Alzheimer’s disease using PET-based Braak staging. Nat Aging. 2022;2(6):526-535. 10.1038/s43587-022-00204-0 PMC10154209 37118445

[42] Shi Y , Zhang W, Yang Y, et al Structure-based classification of tauopathies. Nature. 2021;598(7880):359-363. 10.1038/s41586-021-03911-7 PMC7611841 34588692

[43] Malpetti M , Kaalund SS, Tsvetanov KA, et al In vivo ^18^F-flortaucipir PET does not accurately support the staging of progressive supranuclear palsy. J Nucl Med Off Publ Soc Nucl Med. 2022;63(7):1052-1057. 10.2967/jnumed.121.262985 PMC7612961 34795013

[44] Leuzy A , Chiotis K, Lemoine L, et al Tau PET imaging in neurodegenerative tauopathies-still a challenge. Mol Psychiatry. 2019;24(8):1112-1134. 10.1038/s41380-018-0342-8 PMC6756230 30635637

[45] Gogola A , Minhas DS, Villemagne VL, et al Direct comparison of the tau PET tracers ^18^F-flortaucipir and ^18^F-MK-6240 in human subjects. J Nucl Med Off Publ Soc Nucl Med. 2022;63(1):108-116. 10.2967/jnumed.120.254961 PMC8717175 33863821

[46] Masdeu JC , Pascual B, Fujita M. Imaging neuroinflammation in neurodegenerative disorders. J Nucl Med Off Publ Soc Nucl Med. 2022;63(Suppl 1):45S-52S. 10.2967/jnumed.121.263200 35649654

[47] Chen MK , Guilarte TR. Translocator protein 18 kDa (TSPO): molecular sensor of brain injury and repair. Pharmacol Ther. 2008;118(1):1-17. 10.1016/j.pharmthera.2007.12.004 PMC2453598 18374421

[48] Chen Z , Haider A, Chen J, et al The repertoire of small-molecule PET probes for neuroinflammation imaging: challenges and opportunities beyond TSPO. J Med Chem. 2021;64(24):17656-17689. 10.1021/acs.jmedchem.1c01571 PMC9094091 34905377

[49] Martinez-Perez DA , McGlothan JL, Rodichkin AN, et al Amyloid-β plaque-associated microglia drive TSPO upregulation in Alzheimer’s disease. Acta Neuropathol (Berl). 2025;150(1):6. 10.1007/s00401-025-02912-4 PMC12271292 40676304

[50] Wang C , Keliher E, Zeller MWG, et al An activatable PET imaging radioprobe is a dynamic reporter of myeloperoxidase activity in vivo. Proc Natl Acad Sci U S A. 2019;116(24):11966-11971. 10.1073/pnas.1818434116 PMC6575581 31123149

[51] Bavarsad MS , Grinberg LT. SV2A PET imaging in human neurodegenerative diseases. Front Aging Neurosci. 2024;16:1380561. 10.3389/fnagi.2024.1380561 PMC11064927 38699560

[52] Finnema SJ , Nabulsi NB, Eid T, et al Imaging synaptic density in the living human brain. Sci Transl Med. 2016;8(348):348ra96. 10.1126/scitranslmed.aaf6667 27440727

[53] Drake LR , Wu Y, Naganawa M, et al First-in-human study of ^18^F-SynVesT-2: an SV2A PET imaging probe with fast brain kinetics and high specific binding. J Nucl Med Off Publ Soc Nucl Med. 2024;65(3):462-469. 10.2967/jnumed.123.266470 PMC10924160 38360052

[54] Chen MK , Mecca AP, Naganawa M, et al Comparison of [11C]UCB-J and [18F]FDG PET in Alzheimer’s disease: a tracer kinetic modeling study. J Cereb Blood Flow Metab Off J Int Soc Cereb Blood Flow Metab. 2021;41(9):2395-2409. 10.1177/0271678X211004312 PMC8393289 33757318

[55] Giorgio J , Soleimani-Meigooni DN, Janabi M, et al Imaging synaptic density in aging and Alzheimer disease with [18F]SynVesT-1. J Nucl Med Off Publ Soc Nucl Med. 2025;66(4):620-625. 10.2967/jnumed.124.269005 PMC11960604 40049743

[56] Andersen KB , Hansen AK, Schacht AC, et al Synaptic density and glucose consumption in patients with Lewy body diseases: an [11 C]UCB-J and [18 F]FDG PET study. Mov Disord Off J Mov Disord Soc. 2023;38(5):796-805. 10.1002/mds.29375 36905188

[57] Malpetti M , Jones PS, Cope TE, et al Synaptic loss in frontotemporal dementia revealed by [11 C]UCB-J positron emission tomography. Ann Neurol. 2023;93(1):142-154. 10.1002/ana.26543 PMC10099663 36321699

[58] Kumakura Y , Cumming P. PET studies of cerebral levodopa metabolism: a review of clinical findings and modeling approaches. Neurosci Rev J Bringing Neurobiol Neurol Psychiatry. 2009;15(6):635-650. 10.1177/1073858409338217 19793723

[59] Ibrahim N , Kusmirek J, Struck AF, et al The sensitivity and specificity of F-DOPA PET in a movement disorder clinic. Am J Nucl Med Mol Imaging. 2016;6(1):102-109. PMC4749509 27069770

[60] Thobois S , Prange S, Scheiber C, Broussolle E. What a neurologist should know about PET and SPECT functional imaging for parkinsonism: a practical perspective. Parkinsonism Relat Disord. 2019;59:93-100. 10.1016/j.parkreldis.2018.08.016 30181086

[61] Smith R , Capotosti F, Schain M, et al The α-synuclein PET tracer [18F] ACI-12589 distinguishes multiple system atrophy from other neurodegenerative diseases. Nat Commun. 2023;14(1):6750. 10.1038/s41467-023-42305-3 PMC10611796 37891183

[62] Pees A , Grotegerd AK, Bleher D, Herfert K, Vasdev N. PET imaging of alpha-synuclein: from radiotracer design through in vitro and in vivo translation. Eur J Nucl Med Mol Imaging. 2026;53(6):4211-4239. 10.1007/s00259-025-07695-0 PMC13121216 41559331

[63] Hatano T , Lin CH, Cardoso F. A Biological framework for Parkinson’s disease: advances in α-synuclein-centered biomarkers and staging. J Neurochem. 2026;170(6):e70491. 10.1111/jnc.70491 PMC13542848 42261981

[64] Currie G. Molecular theranostics: principles, challenges and controversies. J Med Radiat Sci. 2025;72(1):156-164. 10.1002/jmrs.836 PMC11909690 39485717

[65] Kevadiya BD , Ottemann BM, Thomas MB, et al Neurotheranostics as personalized medicines. Adv Drug Deliv Rev. 2019;148:252-289. 10.1016/j.addr.2018.10.011 PMC6486471 30421721

[66] Klein G , Rabinovici GD, Zetterberg H, et al Interim biomarker results for trontinemab, a novel Brainshuttle^TM^ antibody in development for the treatment of Alzheimer’s disease. Alzheimers Dement. 2025;21(S5):e104288. 10.1002/alz70859_104288

[67] Chaparro CIP , Simões BT, Borges JP, Castanho MARB, Soares PIP, Neves V. A promising approach: magnetic nanosystems for Alzheimer’s disease theranostics. Pharmaceutics. 2023;15(9):2316. 10.3390/pharmaceutics15092316 PMC10536416 37765284

[68] McCluskey SP , Plisson C, Rabiner EA, Howes O. Advances in CNS PET: the state-of-the-art for new imaging targets for pathophysiology and drug development. Eur J Nucl Med Mol Imaging. 2020;47(2):451-489. 10.1007/s00259-019-04488-0 PMC6974496 31541283

[69] Chen B , Marquez-Nostra B, Belitzky E, et al PET imaging in animal models of Alzheimer’s disease. Front Neurosci. 2022;16:872509. 10.3389/fnins.2022.872509 PMC9171374 35685772

[70] Josephson L , Rudin M. Barriers to clinical translation with diagnostic drugs. J Nucl Med Off Publ Soc Nucl Med. 2013;54(3):329-332. 10.2967/jnumed.112.107615 23359658

